# GABA_A_ Receptors and Drug-Resistant Epilepsy: Why It’s Time to Think Outside the Pore

**DOI:** 10.1007/s10571-026-01759-x

**Published:** 2026-06-04

**Authors:** Iman Imtiyaz Ahmed Juvale, Alina Arulsamy

**Affiliations:** Neuropharmacology Research Laboratory, Jeffrey Cheah School of Medicine and Health Sciences, Monash University Malaysia, 47500 Bandar Sunway, Selangor Malaysia

**Keywords:** Drug-resistant epilepsy, GABA, GABA_A_ receptors, Seizures, Antiseizure medications, Benzodiazepines, Neurosteroids

## Abstract

Drug-resistant epilepsy (DRE) represents a major clinical hazard, characterised by persistent uncontrolled seizures that elevate risks of trauma, cognitive deterioration, psychiatric comorbidity, and sudden unexpected death in epilepsy, while cumulative medication exposure, healthcare burden, and functional impairment substantially worsen long-term morbidity and quality of life. In DRE, disruptions in GABA_A_ receptors (GABA_A_R) function through altered subunit composition, receptor mislocalisation, or impaired trafficking, undermine inhibitory control and contribute to persistent seizures. The regulation of intracellular chloride by NKCC1 and KCC2 further shapes GABA_A_R-mediated inhibition, where imbalances in these transporters exacerbate network hyperexcitability. Structural studies, including high-resolution cryo-EM, reveal how ligand binding, receptor conformation, and interactions with auxiliary proteins modulate GABA_A_R activity, offering insights into why some subtypes respond differently to benzodiazepines or neurosteroids. Emerging evidence suggests that targeting receptor-associated proteins, trafficking pathways, or specific subunit combinations may offer novel ways to restore inhibitory tone and overcome pharmacoresistance. Understanding how these molecular, structural, and cellular factors converge to alter GABA_A_R function provides a roadmap for designing therapies that are both effective and precise, especially for DRE. By unravelling the mechanisms behind GABA_A_R dysfunction in DRE, new avenues open for restoring network stability and improving outcomes for patients living with DRE.

## Introduction

Epilepsy is a devastating neurological disorder with profound global consequences, ranking third in the disability-adjusted life years lost and second only to stroke in potential years of life lost due to sudden unexpected death in epilepsy (SUDEP) (Mesraoua et al. 2022; Sumedha Maturu et al. 2023). With nearly 70 million people affected worldwide, epilepsy knows no boundaries; it strikes across all ages, ethnicities, and regions, with a mortality risk up to ten times higher than that of the general population (Puteikis and Mameniškienė 2021; Trinka et al. 2023). Alarmingly, about 75% of cases begin in childhood, highlighting the brain’s heightened vulnerability to seizures during critical periods of development (Sumadewi et al. 2023). Clinically, epilepsy is more than just seizures; it disrupts consciousness, motor control, sensory perception, mood, cognition, and even survival itself, leaving individuals at risk of life-threatening complications (Sumadewi et al. 2023). While antiseizure medications (ASMs) are the gold-standard treatment for most epilepsy types, approximately 30% of patients develop drug-resistant epilepsy (DRE), defined by persistent seizures despite trials of at least two appropriately chosen and tolerated ASMs, a proportion that has remained largely unchanged for decades because the vast majority of ASMs control symptoms rather than modify underlying pathophysiology (Lagger et al. 2023). The origins of epilepsy are as diverse as its impact, spanning genetic predispositions, structural abnormalities, metabolic dysfunctions, immune responses, and infections. Traumatic brain injuries, strokes, brain infections like meningitis and encephalitis, neurodegenerative diseases, and congenital malformations are all well-documented triggers (Rho and Boison 2022). Yet, despite this variety in causes, all forms of epilepsy converge on a fundamental neurological dysfunction: an imbalance between excitation and inhibition in the brain. In a healthy system, neural activity is finely tuned by the interplay between glutamate, the primary excitatory neurotransmitter, and gamma-aminobutyric acid (GABA), the chief inhibitory counterpart (Sarlo and Holton 2021). In epilepsy, however, this delicate equilibrium collapses, glutamate surges, GABA declines, and neurons spiral into uncontrolled hyperexcitability, resulting in seizures. At the heart of this dysfunction lies the GABA_A_ receptor (GABA_A_R), the most prevalent mediator of inhibitory neurotransmission and a principal target of current therapeutics; impaired GABA_A_R function, altered subunit expression, and associated inhibitory deficits contribute to both ictogenesis and pharmacoresistance, underscoring the excessive excitability that defines epilepsy, making it a central focus for therapeutic strategies (Perucca et al. 2023).

## Structural Organisation and Subunit Diversity of GABA_A_ Receptors

**Fig. 1:**
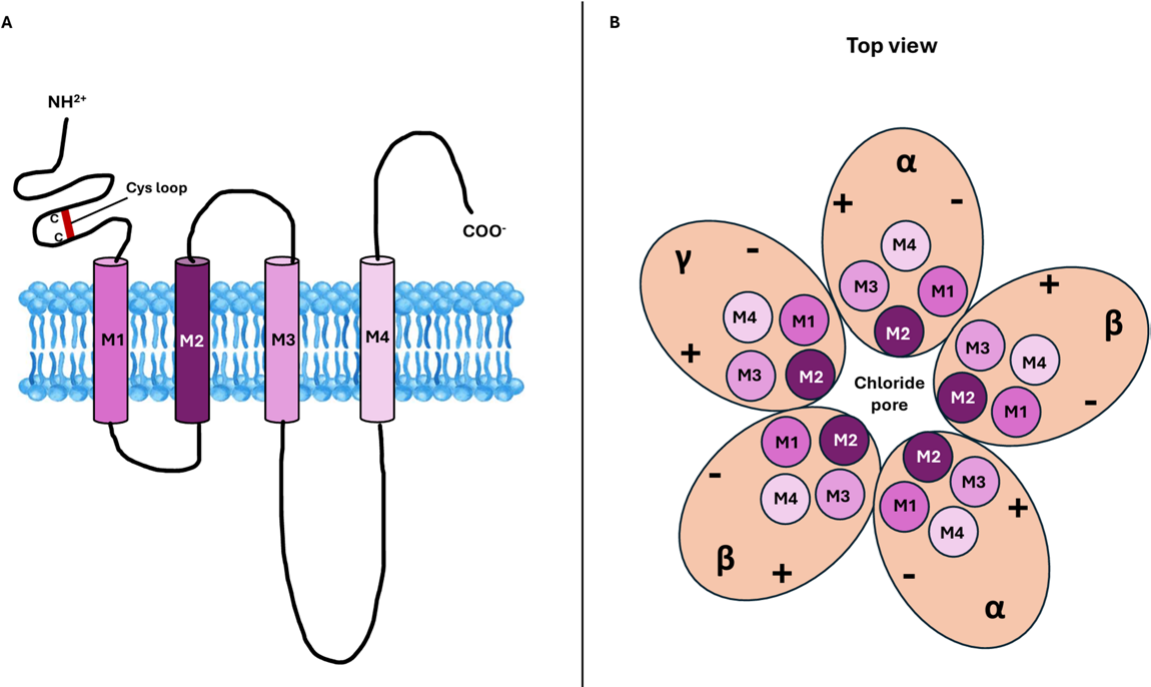
Structural organisation of the GABA_A_ receptor transmembrane domains. **A** Each GABA_A_ receptor subunit contains four membrane-spanning helices (M1–M4) that contribute to receptor stability and ion channel formation. **B** A top-view representation of the pentameric assembly illustrates the radial arrangement of five subunits around the central chloride-conducting pore

GABA is the principal inhibitory neurotransmitter in the adult central nervous system (CNS), and fast synaptic inhibition is mediated primarily through ionotropic GABA_A_Rs (Chuang and Reddy 2018). These receptors are ligand-gated anion channels expressed on postsynaptic membranes that, upon activation, permit the flux of chloride (Cl⁻) and bicarbonate (HCO₃⁻) ions (Raimondo et al. 2017). The direction and magnitude of this flux determine whether GABAergic signalling is inhibitory or, paradoxically, excitatory. This outcome is governed by the intracellular chloride concentration and the resulting reversal potential for GABA_A_Rs (E_GABA_). When intracellular Cl⁻ is low, E_GABA_ is hyperpolarised relative to the resting membrane potential, producing membrane hyperpolarisation and neuronal inhibition (Raimondo et al. 2017). Conversely, elevated intracellular Cl⁻ shifts E_GABA_ toward depolarised values, allowing GABA signalling to become less inhibitory or even excitatory, thereby promoting network hyperexcitability. This condition occurs physiologically in immature neurons during early brain development, where high intracellular chloride concentrations render GABA signalling depolarising rather than inhibitory (Ben-Ari et al. 1989; Mueller et al. 1983). Similar chloride dysregulation can also occur in pathological states such as epilepsy, traumatic brain injury, and certain neurodevelopmental disorders, where impaired chloride cotransporter-mediated chloride extrusion leads to reduced inhibitory efficacy of GABA and increased network hyperexcitability (Dzhala et al. 2012; Jaenisch et al. 2010; Jarvis et al. 2023; Sivakumaran et al. 2015). GABA_A_Rs belong to the Cys-loop ligand-gated ion channel superfamily and exhibit remarkable molecular heterogeneity (Chuang and Reddy 2018). Excluding splice variants, at least 19 subunits have been identified in humans, including six α (α1–6), three β (β1–3), three γ (γ1–3), three ρ (ρ1–3), and one each of δ, ε, θ, and π (Sigel and Steinmann 2012). Each mature subunit (~ 450 amino acids) contains a large extracellular N-terminal domain, four transmembrane helices (M1–M4), and a prominent intracellular loop between M3 and M4 that regulates receptor trafficking, anchoring, and phosphorylation (Sigel and Steinmann 2012). Five subunits assemble into a pentameric complex surrounding a central pore lined by the M2 domains (Fig. 1).

During early CNS development, the GABA_A_Rs exhibit subunit compositions that differ substantially from those observed in the mature brain. Much of the evidence describing these immature receptor configurations comes from rodent models. Developmentally, the brain of a postnatal day 8–10 (PN8–10) rat is considered roughly equivalent to that of a human neonate, while the rat infantile stage (PN7–21) corresponds broadly to early infancy in humans (Zapatero-Caballero et al. 2003, 2004). In the embryonic rat neocortex, GABA is detectable by embryonic day 10 (E10) and becomes spatially restricted by E14 to regions such as the subplate, cortical plate, marginal zone, and intermediate zone (Haydar et al. 2000). Notably, GABA_A_Rs are expressed before the formation of functional GABAergic synapses, enabling GABA to act through autocrine and paracrine mechanisms to regulate neurodevelopmental processes, including neural progenitor proliferation, neuronal migration, differentiation, and survival (Behar et al. 1996; Haydar et al. 2000; Heck et al. 2007; Ikeda et al. 1997; Laurie et al. 1992; Luk and Sadikot 2001; Ma and Barker 1995; Serafini et al. 1998). Immature GABA_A_Rs display region- and cell-type-specific subunit expression. In the ventricular zone, where premigratory neuroblasts and neural progenitor cells reside, receptors commonly contain α4, β1, and γ1 subunits (Laurie et al. 1992; Ma and Barker 1995). In contrast, neurons within the cortical and subcortical plates, including developing glutamatergic projection neurons and immature interneurons, predominantly express α2, α3, β3, and γ2 subunits (Maric et al. 1997). These receptors form pentameric chloride channels typically arranged as two α subunits, two β subunits, and one γ subunit, although the precise combinations are more heterogeneous than in the adult CNS. Functionally, immature receptor assemblies frequently include α2βxγ2 or α3βxγ2 configurations, and in certain neuronal populations, such as hippocampal dentate granule cells, α5-containing receptors are also prominent (Brooks-Kayal et al. 2001; Kanaumi et al. 2006; Kapur and Macdonald 1999; Liu and Wong-Riley 2006; Mtchedlishvili et al. 2003; Ravizza et al. 2003; Rissman et al. 2006; Yu et al. 2006). These subunit compositions produce slower channel kinetics and prolonged inhibitory postsynaptic currents (IPSCs) compared with mature receptors. Although numerous combinations are theoretically possible, receptor composition and arrangement critically determine channel kinetics, subcellular localisation, and pharmacological sensitivity, with the predominant adult CNS isoform consisting of α1β2γ2 subunits arranged in a defined stoichiometry (Rowlett et al. 2005).

## Synaptic and Extrasynaptic Signalling: Phasic and Tonic Inhibition

Functional diversity of GABA_A_Rs arises from both subunit composition and cellular localisation (Table 1). Immunogold electron microscopy in rat cerebellar neurons and patch-clamp recordings in rat hippocampal neurons confirmed that synaptic receptors typically contain α1–3, β1–3, and γ2 subunits and mediate phasic inhibition, producing rapid, spatially precise increases in Cl⁻ conductance that regulate spike timing, dendritic integration, and network synchronisation (Nusser et al. 1996; Sun et al. 2004). In contrast, whole-cell electrophysiological recordings in mouse thalamic relay neurons and hippocampal dentate granule cells, as well as δ-subunit knockout mouse models, show that extrasynaptic GABA_A_Rs frequently incorporate α4–6 and δ subunits and mediate tonic inhibition by detecting low ambient extracellular GABA and generating a persistent inhibitory conductance that regulates baseline neuronal excitability (Caraiscos et al. 2004; Chandra et al. 2006; Glykys et al. 2008). Prominent extrasynaptic isoforms include α4βδ receptors in forebrain regions such as the hippocampus and cortex, and α6βδ receptors in cerebellar granule cells, identified through mouse cerebellar electrophysiology and recombinant receptor expression studies (Goetz et al. 2007). Consistent with these findings, slice electrophysiology in mouse hippocampal CA1 pyramidal neurons demonstrated that tonic GABAergic conductance functions as a shunting inhibitory mechanism that increases the excitatory input required to reach firing threshold, thereby stabilising neuronal network activity (Song et al. 2011). Conversely, α5 GABA_A_Rs are predominantly extrasynaptic, mediating tonic inhibition in hippocampal CA1/CA3 pyramidal neurons and layer 5 cortical neurons, while exhibiting comparatively lower levels of expression in the dentate granule cells, with clustered dendritic localisation demonstrated in various rodent models (Brünig et al. 2002; Crestani et al. 2002; Glykys et al. 2008). They also contribute to phasic sIPSCs, eIPSCs, and slow IPSCs, particularly on dendrites innervated by somatostatin interneurons and neurogliaform cells, generating outward-rectifying currents that regulate NMDA receptor activation and pyramidal excitability (Magnin et al. 2019; Prenosil et al. 2006; Schulz et al. 2018). Human and mouse cortex show α5 enrichment in pyramidal and parvalbumin cells (Hu et al. 2019). Gabra5−/− and α5H105R mice reveal that α5 reduction diminishes tonic/phasic currents, gamma oscillations, long term potentiation, and hippocampal-dependent learning, establishing α5 GABA_A_Rs as critical dendritic inhibitory regulators (Collinson et al. 2002; Crestani et al. 2002; Martin et al. 2010; Towers et al. 2004).

**Table 1:** A summary of GABA_A_ receptor subunits: function, distribution, and pharmacological relevance

Subunit	Function	Localization	Drug interaction	Experimental model	References
**α1**	The α1 subunit mediates fast phasic synaptic inhibition and is primarily responsible for the sedative and anticonvulsant actions of GABA_A_ receptors.	It is widely expressed throughout the cortex, thalamus, cerebellum, and hippocampus.	Receptors containing α1 show high sensitivity to benzodiazepines and are also modulated by hypnotics such as zolpidem and anaesthetics, including propofol.	Bovine brain cDNA cloning; Xenopus oocyte electrophysiology; α_1_H101R mutant mice; rat brain cDNA cloning	(Khrestchatisky et al. 1989; McKernan et al. 2000; Schofield et al. 1987)
**α2**	The α2 subunit contributes to anxiolysis and seizure suppression by regulating inhibitory tone in emotional and limbic circuits.	It is predominantly located in the hippocampus, amygdala, and other limbic regions.	α2-containing receptors are sensitive to benzodiazepines and mediate much of their anxiolytic and anticonvulsant pharmacology.	Rat brain cDNA cloning; heterologous receptor expression; knockout mice (Gabra2^tm2.2Uru^)	(Lolait et al. 1989; Marksitzer et al. 1993; Vollenweider et al. 2011)
**α3**	The α3 subunit regulates arousal and motor control by modulating synaptic inhibition.	It is enriched in the reticular thalamus, brainstem, and cortical regions.	Receptors incorporating α3 respond to benzodiazepines and contribute to muscle-relaxant and sedative drug effects.	Rat brain cDNA library cloning and sequencing; behavioural assessment	(Atack et al. 2005; Dias et al. 2005; Levitan et al. 1988; Malherbe et al. 1990; Mertens et al. 1993)
**α4**	The α4 subunit primarily mediates extrasynaptic tonic inhibition and regulates neuronal excitability rather than fast synaptic transmission.	It is highly expressed in the dentate gyrus, thalamus, and cortical areas.	α4-containing receptors are insensitive to benzodiazepines but are strongly modulated by neurosteroids and agonists such as gaboxadol, and they are upregulated in drug-resistant epilepsy.	Human brain cDNA cloning; recombinant receptor expression and ligand binding assays; α4 knockout mice; rat hippocampal electrophysiology; rat brain cDNA cloning	(Khrestchatisky et al. 1989; Wafford et al. 1996; Yang et al. 1995; Sanie Ymer et al. 1989a, b)
**α5**	The α5 subunit regulates tonic inhibition, which is involved in learning and memory processing within hippocampal circuits.	It is predominantly localised to pyramidal neurons in the CA1–CA3 regions of the hippocampus.	α5-containing receptors are sensitive to benzodiazepines; chronic expression leads to diazepam tolerance, and inverse agonists targeting this subunit can alter cognitive performance.	Mice hippocampal transcriptome analysis, behavioural studies, quantitative receptor audioradiography	(Prut et al. 2010; van Rijnsoever et al. 2004; Wang et al., 2012, 2024)
**α6**	The α6 subunit shapes cerebellar inhibitory signalling and motor coordination by controlling granule cell excitability.	It is selectively expressed in cerebellar granule cells.	Receptors containing α6 are insensitive to benzodiazepines but are modulated by neurosteroids and ethanol.	Human cerebellum cDNA cloning; receptor expression in oocytes; rat brain cDNA cloning	(Khrestchatisky et al. 1989; Lüddens et al. 1990; Wallner et al. 2014)
**β1**	The β1 subunit contributes to channel gating and determines the kinetics of GABA_A_ receptor currents.	It is broadly distributed throughout the central nervous system.	β1-containing receptors provide binding sites for anaesthetics such as propofol and barbiturates.	Bovine brain cDNA cloning; Xenopus oocyte electrophysiology	(Schofield et al. 1987; Yanovsky et al. 2012; Sanie Ymer et al. 1989a, b)
**β2**	The β2 subunit controls conductance and receptor assembly, thereby stabilising inhibitory signalling.	It is expressed in the cortex, hippocampus, and thalamus.	β2 subunits are targets for general anaesthetics, and mutations in this subunit have been linked to epilepsy phenotypes.	Rat brain cDNA cloning and recombinant receptor expression; β2 N265S mutant mice	(Benke et al. 1991a; Reynolds et al. 2003; Ymer et al. 1989a, b)
**β3**	The β3 subunit is critical for maintaining inhibitory tone and regulating neuronal excitability.	It is abundant in the hippocampus, cortex, and thalamus.	β3 provides major binding sites for anaesthetics such as propofol and etomidate, and genetic variants are associated with epilepsy syndromes.	Rat brain cDNA cloning; heterologous receptor expression	(Shi et al. 2019; Yanovsky et al. 2012; Ymer et al. 1989a, b)
**γ1**	The γ1 subunit participates in synaptic targeting and modulation of receptor function.	It is expressed in cortical and hippocampal neurons.	γ1-containing receptors show reduced but present sensitivity to benzodiazepines compared with γ2-containing receptors.	cDNA cloning and recombinant receptor expression	(Khom et al. 2006; Ymer et al. 1990)
**γ2**	The γ2 subunit anchors GABA_A_ receptors at synapses and enables fast inhibitory neurotransmission.	It is widely distributed in cortical, hippocampal, and cerebellar neurons.	γ2 is essential for benzodiazepine sensitivity, and mutations disrupt benzodiazepine efficacy and are associated with genetic epilepsies.	Recombinant receptors expressed in mammalian cells and electrophysiology; rat brain cDNA cloning	(Khrestchatisky et al. 1989; Pritchett et al. 1989; Shivers et al. 1989)
**γ3**	The γ3 subunit contributes to developmental and modulatory aspects of GABAergic signalling.	It is localised in the thalamic and cortical regions.	γ3-containing receptors show limited pharmacological targeting and minor benzodiazepine sensitivity.	cDNA cloning and sequence analysis	(Knoflach et al. 1991; Richter et al. 2020)
**δ**	The δ subunit mediates persistent tonic inhibition that strongly controls neuronal excitability outside synapses.	It is localised mainly to the dentate gyrus, thalamus, and cerebellum.	δ-containing receptors are insensitive to benzodiazepines but highly sensitive to neurosteroids and gaboxadol, making them attractive targets for non-classical antiseizure strategies.	Rat brain cDNA cloning and expression analysis	(Benke et al. 1991b; Shivers et al. 1989; Wafford et al. 2009)
**ε**	The ε subunit alters receptor gating and contributes to region-specific inhibitory signalling.	It is found in the brainstem and hypothalamus.	ε-containing receptors exhibit low sensitivity to benzodiazepines and modify anaesthetic responses.	Human brain cDNA cloning and expression systems	(Neelands et al. 1999; Ranna et al. 2006)
**θ**	The θ subunit contributes to receptor assembly and functional modulation of inhibitory currents.	It is expressed in cortical and hippocampal regions.	θ-containing receptors are minimally targeted by current antiseizure drugs.	Human brain cDNA cloning and sequence analysis	(Bonnert et al. 1999; WHITING et al. 1999)
**π**	The π subunit is involved in non-neuronal and specialised inhibitory signalling.	It is predominantly expressed in peripheral tissues, such as the uterus and lungs, with limited expression in the central nervous system.	π-containing receptors have little relevance for conventional benzodiazepine or antiseizure drug targeting.	Human tissue cDNA cloning and expression analysis	(Fujii and Mellon 2001; Hedblom and Kirkness 1997; Jin et al. 2006)
**ρ1**	The ρ1 subunit forms GABA_C_ receptors that generate slow, sustained inhibitory currents distinct from those of classical GABA_A_ receptors.	It is mainly expressed in the retina and serves spinal cord nociceptive functions.	ρ1-containing receptors are insensitive to benzodiazepines and barbiturates and are pharmacologically distinct from classical GABA_A_ receptors.	Human retina cDNA cloning; electrophysiology in expression systems; knockout mice (rho1^−/−^)	(Ekema et al. 2002; Schlicker et al. 2009; Zheng et al. 2003)
**ρ2**	The ρ2 subunit contributes to visual signal processing through prolonged inhibitory signalling.	It is expressed in retinal neurons.	ρ2-containing receptors are insensitive to benzodiazepines and show unique pharmacology compared with GABA_A_ receptors.	Human retinal cDNA cloning and recombinant receptor expression	(Boue-Grabot et al. 1998; Enz and Cutting 1999; Wang et al. 1994)
**ρ3**	The ρ3 subunit participates in developmental and visual inhibitory pathways.	It is found in the retina and superior colliculus.	ρ3-containing receptors show limited relevance for conventional benzodiazepine-based therapeutics.	Human retinal cDNA cloning and functional expression	(Alakuijala et al. 2005; Ogurusu and Shingai 1996)

## Chloride Homeostasis and the Polarity of GABAergic Transmission

**Fig. 2:**
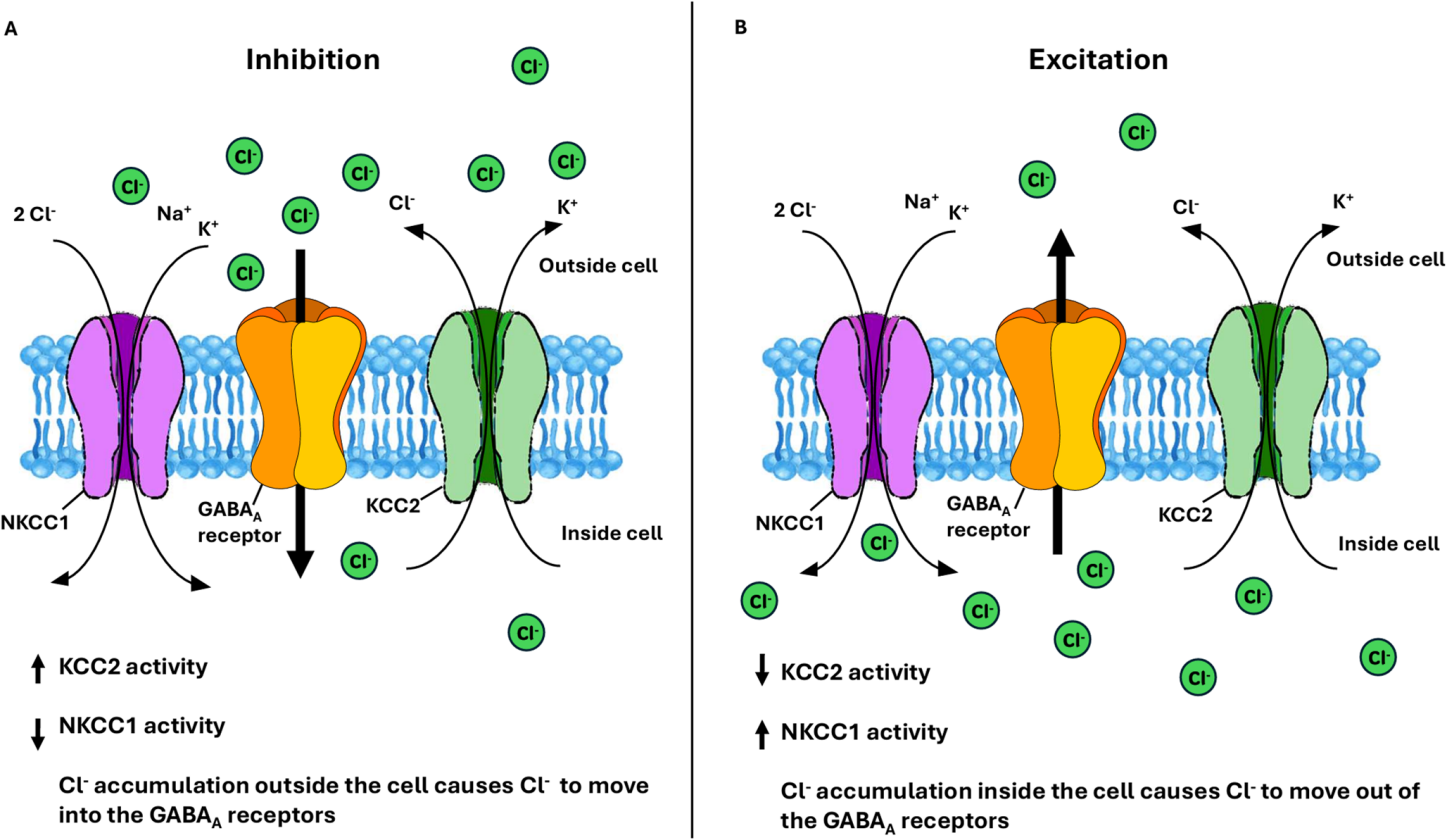
Chloride homeostasis determines whether GABA_A_ receptor signalling is inhibitory or excitatory. **A** Under physiological conditions, high activity of the potassium–chloride cotransporter KCC2 extrudes chloride ions from neurons, maintaining a low intracellular chloride concentration. Activation of GABA_A_ receptors therefore drives the membrane to hyperpolarise and produces inhibitory synaptic signalling. **B** When chloride regulation is disrupted, increased activity of the sodium–potassium–chloride cotransporter NKCC1 elevates intracellular chloride levels. In this state, the opening of GABA_A_ receptors leads to chloride efflux, membrane depolarisation, and paradoxically, excitatory GABAergic signalling

The inhibitory efficacy of GABA_A_R activation depends fundamentally on intracellular chloride regulation. Chloride homeostasis is maintained primarily by two opposing cotransporters belonging to the solute carrier family 12 (*SLC12*): Na⁺–K⁺–Cl⁻ cotransporter 1 (NKCC1; encoded by *SLC12A2*) and K⁺–Cl⁻ cotransporter 2 (KCC2; encoded by *SLC12A5*) (Lam et al. 2023). NKCC1 cotransports one Na⁺, one K⁺, and two Cl⁻ ions into neurons, whereas KCC2 extrudes K⁺ and Cl⁻ in a 1:1 ratio (Fig. 2).

During early development, NKCC1 predominates, resulting in elevated intracellular Cl⁻ and a depolarised E_GABA_, such that GABA_A_R activation is excitatory (Ben-Ari et al. 1989; Delpy et al. 2008). As neurons mature, KCC2 expression increases, lowering intracellular Cl⁻ and shifting E_GABA_ to hyperpolarised values, producing the classical excitatory-to-inhibitory developmental switch in GABAergic signalling that is essential for synaptic maturation and circuit formation (Cancedda et al. 2007). Genetic disruption of NKCC1 delays the development of both GABAergic and glutamatergic synapses, underscoring its importance in early network organisation (Pfeffer et al. 2009). At the molecular level, KCC2 function is tightly regulated by phosphorylation. Phosphorylation of serine 940 enhances KCC2 membrane stability and transport activity, whereas phosphorylation of threonine 906 and 1007 suppresses transporter function (Kahle et al. 2013). These processes are controlled in part by the with-no-lysine (WNK) kinase pathway, which regulates both NKCC1 and KCC2. Through this mechanism, neurons dynamically tune intracellular Cl⁻ and thereby determine the polarity and strength of GABAergic inhibition. In adults, under physiological conditions, a low intracellular Cl⁻ concentration maintains a negative E_GABA_, such that activation of GABA_A_R produces Cl⁻ influx and membrane hyperpolarisation, thereby increasing the excitatory input required to reach the action potential threshold. Even when E_GABA_ shifts modestly toward depolarised values but remains below the firing threshold, GABAergic signalling can still exert shunting inhibition by reducing membrane resistance, thereby attenuating excitatory postsynaptic potentials (Edwards 1990; Egawa and Fukuda 2013; Staley and Mody 1992). Consequently, both hyperpolarising and mildly depolarising GABAergic responses generally maintain an inhibitory influence on neuronal excitability. In conditions such as DRE, however, intense and repetitive activation of GABAAR during epileptiform activity drives rapid intracellular Cl⁻ accumulation, progressively depolarising E_GABA_ (Fujiwara-Tsukamoto et al. 2010; Ilie et al. 2012; Isomura et al. 2003; Lamsa and Kaila 1997). This activity-dependent chloride loading increases with the strength of GABAergic activation and gradually erodes GABA_A_R–mediated inhibition, thereby promoting network hyperexcitability (Ilie et al. 2012; Lasztóczi et al. 2009; Lillis et al. 2012; Lopantsev and Avoli 1998). As seizure pathology progresses, prolonged seizures, such as status epilepticus, induce internalisation and functional loss of postsynaptic GABA_A_Rs, markedly reducing inhibitory synaptic transmission and contributing to persistent, pharmacoresistant seizure activity (Kapur and Macdonald 1997; Mazarati et al. 1998; Treiman et al. 1998).

## Remodelling of GABA_A_ Receptors in Drug-Resistant Epilepsy

**Fig. 3:**
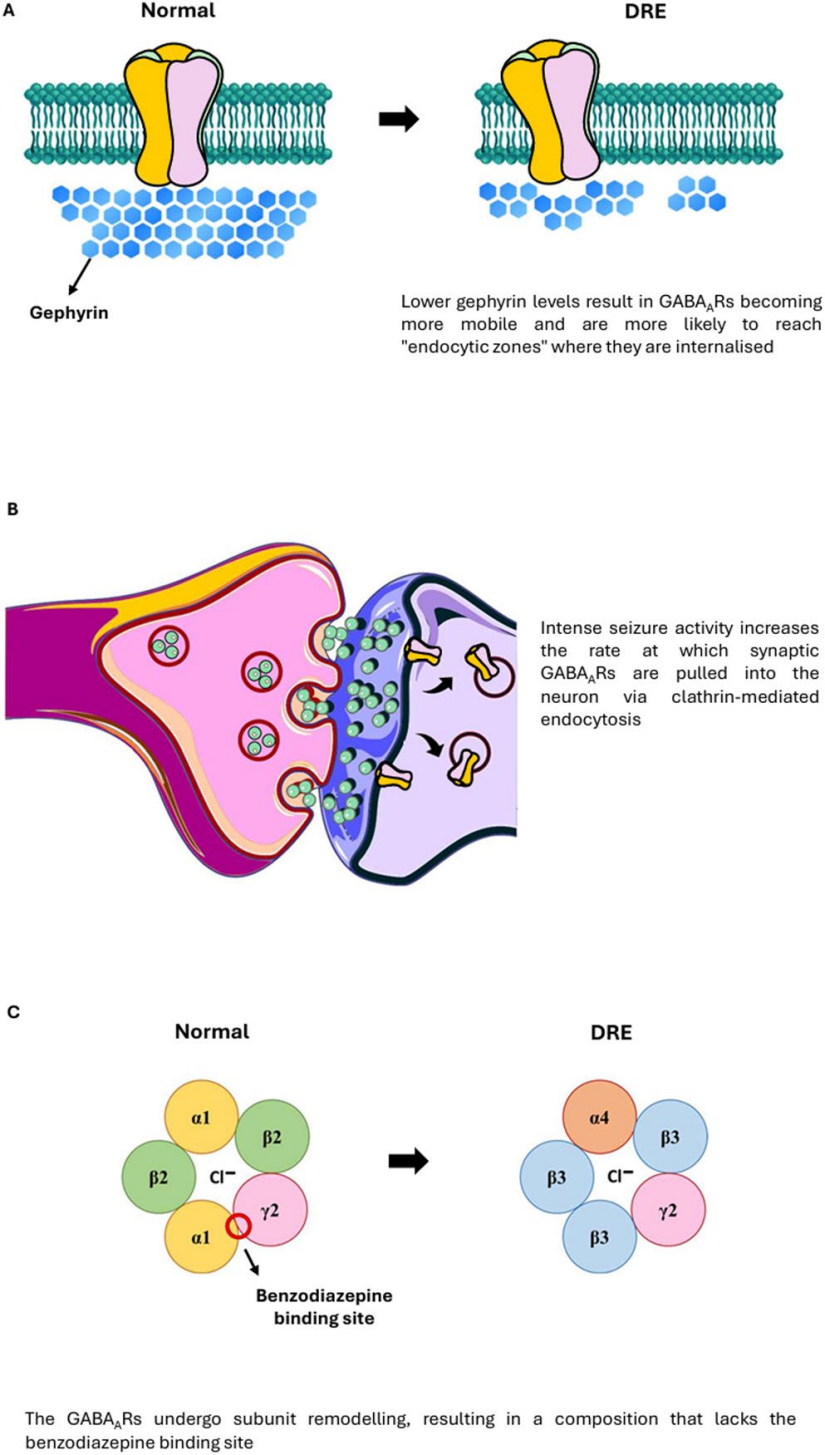
Alterations in GABA_A_ receptors in DRE. **A** Under physiological conditions, gephyrin stabilizes synaptic GABA_A_ receptors by anchoring them at the postsynaptic membrane, thereby maintaining receptor clustering and limiting lateral diffusion; **B** Reduced gephyrin expression and disrupted trafficking in DRE lead to increased receptor internalisation and decreased surface expression, resulting in reduced GABA binding and impaired inhibitory neurotransmission; **C** Altered subunit composition, typically involving a shift from α1β2γ2 to α4-containing assemblies, leads to the loss of the α^+^/γ^−^ benzodiazepine binding interface (shown with a red circle), contributing to reduced sensitivity to benzodiazepines such as diazepam

The International League Against Epilepsy (ILAE) defines DRE as the failure to achieve sustained seizure freedom following adequate trials of two appropriately selected, well-tolerated, and properly administered antiseizure medication (ASM) regimens, whether as monotherapies or in combination (Kwan et al. 2010). GABA_A_R subunit composition dictates pharmacological responsiveness. Benzodiazepines bind at the α–γ interface (Fig. 3C), and their effects depend on α-subunit identity. α1-containing receptors mediate sedative and antiseizure effects, whereas α2- and α3-containing receptors contribute to anxiolytic and myorelaxant actions (Rowlett et al. 2005). In contrast, α4- and α6-containing receptors are insensitive to classical benzodiazepines due to a critical arginine substitution within the binding site (Benson et al. 1998). Consequently, ASMs that rely on GABA_A_R modulation lose effectiveness when receptor composition shifts toward α4-containing assemblies (Chaudhuri and Hosur 2024; Sharma et al. 2021).

In pilocarpine- and kainate-induced status epilepticus models, as well as kindling models, benzodiazepine efficacy is markedly reduced: clonazepam shows diminished inhibition in CA1 pyramidal neurons, while valproate and phenobarbital fail to suppress epileptiform firing in CA3 (Brooks-Kayal et al. 1998; Gibbs et al. 1997; Klitgaard et al. 2003). This loss of sensitivity correlates with seizure-driven replacement of α1-containing GABA_A_Rs with α4-containing assemblies, reducing classical benzodiazepine modulation, while intense seizure activity also promotes clathrin-mediated endocytosis and internalisation of synaptic GABA_A_Rs, further diminishing inhibitory neurotransmission (Fig. 3B). In CA1 neurons from these models, α4βXγ2 receptors localise perisynaptically, exhibiting increased tonic conductance but diminished pharmacological responsiveness (Farrant and Nusser 2005; Jacob et al. 2008; Leidenheimer 2008). Rodent genetic models further highlight the functional importance of extrasynaptic inhibition: deletion of δ- or α5-containing GABA_A_Rs increases seizure susceptibility, demonstrating the role of tonic inhibition in stabilising network excitability (Glykys et al. 2008). Reductions in α5 and δ subunits in these models compromise both tonic and shunting inhibition, promoting hyperexcitability and seizure propagation (Kammel et al. 2018).

Clinical studies corroborate findings from animal models that epilepsy is accompanied by extensive molecular and functional remodelling of GABA_A_Rs, particularly those mediating tonic inhibition (Castro-Torres et al. 2020; Loup et al. 2000; Pirker et al. 2003; Sperk et al. 2021). Analyses of human resected epileptic tissue consistently reveal alterations in the expression, assembly, and subcellular localisation of multiple GABA_A_R subunits, including extrasynaptic receptor populations. Notably, increased expression of the α5 subunit, which mediates tonic inhibition in CA1 pyramidal neurons, and the δ subunit, which controls tonic conductance in dentate granule cells, has been reported in temporal lobe epilepsy (TLE) (Sperk et al. 2021). These findings indicate that tonic GABAergic inhibition is not abolished in epilepsy but is instead preserved and reorganised, positioning extrasynaptic GABA_A_Rs as key regulators of epileptic network dynamics. Immunocytochemical studies further demonstrate the profound reorganisation of synaptic GABA_A_R subunits in human epileptic hippocampus. In resected tissue from mesial and non-mesial TLE patients, altered distribution of α1, α2, α3, β2, β3, and γ2 subunits was observed relative to controls (Loup et al. 2000). In sclerotic CA1 regions, marked reductions in α1-, α3-, β3-, and γ2-subunit immunoreactivity accompanied neuronal loss, whereas non-sclerotic specimens showed comparatively preserved expression. In contrast, β-subunit immunoreactivity was increased throughout the hippocampal formation, particularly in the molecular layer of the dentate gyrus and subiculum, where α3 and γ2 were also overexpressed (Loup et al. 2000). In situ hybridisation studies demonstrate elevated β2 and β3 mRNA in dentate granule cells irrespective of hippocampal sclerosis, indicating coordinated transcriptional and post-translational remodelling of inhibitory receptors (Pirker et al. 2003). Another study noted upregulation of the α2, α4, α5, β3, and γ1 subunits in the cortex of patients with pharmacoresistant TLE (Castro-Torres et al. 2020). Importantly, α4- and α5-containing receptors preferentially mediate tonic inhibition and are relatively insensitive to benzodiazepines, suggesting that seizure activity drives potentially maladaptive reorganisation of GABA_A_Rs toward extrasynaptic, pharmacoresistant configurations (Farrant and Nusser 2005). Thus, pharmacoresistant TLE is characterised by dynamic remodelling of inhibitory receptor architecture rather than uniform loss of inhibition. Related but distinct patterns of GABA_A_R dysregulation also emerge in focal cortical dysplasia (FCD), a major cause of paediatric DRE (Najm et al. 2022). FCD is marked by architectural abnormalities and functionally “immature” neurons within dysplastic cortex, where abnormal synaptic transmission promotes epileptogenesis (Hanai et al. 2010). In this setting, disrupted GABAergic signalling becomes paradoxically pro-epileptogenic when inhibitory transmission fails to restrain excitability and instead facilitates epileptiform synchronisation (Blauwblomme et al. 2019). Reduced expression of β1, together with decreases in α1, α2, and β2 subunits, has been reported in dysplastic and heterotopic neurons (Crino et al. 2001). Whereas FCD showed reduced expression of several synaptic subunits, resected hippocampal tissue from TLE patients more often displayed upregulation of α2, α3, α5, β1–3, γ2, and δ, highlighting pathology- and region-specific strategies of inhibitory remodelling.

Beyond receptor composition, the polarity of GABAergic transmission is critically shaped by intracellular chloride homeostasis. Dysregulation of NKCC1 and KCC2 expression shifts the GABA reversal potential E_GABA_ toward depolarised values, generating excitatory GABAergic transmission in epilepsy (Ben-Ari 2012; Kaila et al. 2014). Inflammatory signalling further amplifies this process. IL-1β alters functional expression of chloride cotransporters, thereby weakening inhibitory strength, while compensatory upregulation of IL-1Ra in dysplastic cortex appears insufficient to counterbalance neuroinflammation (Alfano et al. 2022; Iori et al. 2016; Pozzi et al. 2020). Persistent inflammatory signalling, therefore, converges with transporter dysregulation to destabilise inhibitory control and promote hyperexcitability. During early brain development, high NKCC1 and low KCC2 render GABA excitatory and trophic. Similar profiles are re-expressed in pathological conditions, including tuberous sclerosis, Dravet syndrome, and Rett syndrome (Ruffolo et al. 2016, 2018, 2020; Talos et al. 2012; Tang et al. 2016). In malformed FCD cortex, this immature phenotype may allow aberrant GABAergic signalling to actively promote epileptogenesis rather than suppress it. Other elements of the GABA system are also altered in epilepsy. Although early work suggested preserved GABA transport (Gaspary et al. 1998; Wu et al. 2003, 2007), later studies revealed spatially selective reductions in GABA transporter expression. Decreased GAT-1 and GAT-3 expression is observed in severe hippocampal sclerosis, with a substantial reduction in GAT-3 protein, indicating impaired GABA clearance and a reshaping of inhibitory tone (Schijns et al. 2015). Importantly, GABAergic alterations may initially represent compensatory attempts to restrain hyperexcitability but can become maladaptive. In damaged subiculum, a subset of pyramidal neurons responds to GABA with depolarisation rather than hyperpolarisation, generating intrinsic epileptiform activity and acting as pacemaker cells for interictal synchronisation (Cohen et al. 2002). Consistent with this, DRE is associated with NKCC1 upregulation and KCC2 downregulation, promoting excitatory GABA actions and weakening inhibitory control (Liu et al. 2020).

## Targeting GABA_A_ Receptors Beyond Classical Benzodiazepines

Because DRE emerges from the convergence of multiple interacting pathogenic mechanisms, including receptor remodelling, disrupted chloride gradients, inflammation, and maladaptive network plasticity, overcoming pharmacoresistance remains a major clinical challenge. In many patients, these alterations coexist within the same epileptogenic network, limiting the efficacy of conventional ASMs. Consequently, therapeutic development has increasingly shifted toward compounds that modulate GABAergic neurotransmission through non-classical mechanisms, particularly those targeting extrasynaptic GABA_A_R and tonic inhibition. Among these approaches, cannabidiol (CBD) has attracted considerable attention following its clinical success in severe podiatric epilepsies such as Dravet syndrome, Lennox–Gastaut syndrome (LGS), and infantile spasms as seen in clinical observational and interventional studies in paediatric epilepsy cohorts (Berg et al. 2024; Bonanni et al. 2023; Koo et al. 2020). Although CBD acts through multiple molecular pathways, it has been shown to function as a positive allosteric modulator of α-containing GABA_A_Rs, with preferential efficacy at α2β2/3γ2L assemblies relative to β1-containing receptors (Bakas et al. 2017; Ruffolo et al. 2018). Importantly, CBD does not engage the classical benzodiazepine binding site, conferring a pharmacological profile distinct from conventional GABAergic drugs. This selectivity may underlie both its anticonvulsant and anxiolytic properties, as well as its capacity to partially restore physiological inhibitory signalling in pharmacoresistant circuits. Clinical trials support modest but clinically meaningful efficacy. In a randomised, double-blind, placebo-controlled study in Dravet syndrome (NCT02091375), adjunctive CBD (20 mg/kg/day) reduced seizure frequency by approximately 50% compared with 27% for placebo, although complete seizure freedom remained uncommon (Devinsky et al. 2019). Similarly, in LGS (NCT02224690), CBD achieved a 44% reduction in seizure frequency versus 22% with placebo (French et al. 2017). Notably, CBD exhibits poor oral bioavailability and extensive first-pass metabolism, and its therapeutic profile is complicated by pharmacokinetic and pharmacodynamic interactions with clobazam, which may augment both efficacy and adverse effects (Gunning et al. 2021; Millar et al. 2020). Nevertheless, its receptor selectivity distinguishes CBD from benzodiazepines and may be particularly relevant in networks that have lost classical benzodiazepine sensitivity.

Endogenous neuroactive steroids (neurosteroids) represent another mechanistically appealing class of GABAergic modulators. Synthesised by neurons and glia, neurosteroids regulate neuronal excitability by modulating GABA_A_Rs via autocrine and paracrine mechanisms (MacKenzie and Maguire 2013). At nanomolar concentrations, they act as positive allosteric modulators through binding sites within the transmembrane domains of α and β subunits and preferentially potentiate δ- and α6-containing extrasynaptic receptors (Treviño et al. 2025). By enhancing tonic inhibition, neurosteroids reduce neuronal gain and network synchronisation, thereby stabilising hyperexcitable circuits. Allopregnanolone (brexanolone, BXN), a progesterone metabolite, robustly potentiates GABA-evoked currents and retains activity at extrasynaptic receptors even when synaptic receptors are downregulated during prolonged seizures (Rogawski et al. 2013). Early phase I/II trials in super-refractory status epilepticus (SE) suggested benefit, with many patients successfully withdrawn from anaesthetic anticonvulsants (Kanes et al. 2016). However, a subsequent phase III randomised trial failed to demonstrate superiority over placebo, underscoring the translational gap between mechanistic promise and durable clinical efficacy in advanced pharmacoresistant states (Rosenthal et al. 2017). To overcome the limitations of endogenous neurosteroids, synthetic analogues such as ganaxolone (GNX) were developed. GNX is a 3β-methylated derivative of allopregnanolone that prevents metabolic back-conversion and eliminates hormonal side effects (Miziak et al. 2020). It positively modulates both synaptic and extrasynaptic GABA_A_Rs, enhancing phasic and tonic inhibition without engaging nuclear steroid receptors or readily producing tolerance. Early proof-of-concept trials in refractory focal epilepsy showed improved seizure control relative to placebo, although statistical power was limited (Laxer et al. 2000). More recently, a phase III trial demonstrated that GNX (1800 mg/day) reduced seizure frequency by ~ 21% compared with ~ 10% for placebo, with good tolerability (Lappalainen et al. 2017). Subgroup analyses further suggest that the most pharmacoresistant patients may derive disproportionate benefit, positioning GNX as a rational adjunctive therapy exploiting tonic inhibition to stabilise epileptic networks. Several newer ASMs also interact with GABA_A_Rs via distinct mechanisms. Stiripentol, approved for Dravet syndrome, prolongs channel opening in a barbiturate-like manner and preferentially modulates α3- and δ-containing receptors (Fisher 2009; Quilichini et al. 2006). Cenobamate, recently approved for focal epilepsy, combines voltage-gated sodium channel blockade with non-benzodiazepine positive allosteric modulation of α1–6-containing GABA_A_Rs, achieving notable seizure freedom rates in clinical trials (Aboumatar et al. 2022; Chung et al. 2020; Krauss et al. 2020; Nakamura et al. 2019; Rosenfeld et al. 2021). In randomized Phase II studies, doses of 200–400 mg/day produced greater than 50% reductions in seizure frequency, with notable seizure-freedom rates of 11–26% during maintenance. Post-hoc analysis indicated that these responder and seizure-free effects were sustained beyond 12 months. Consistent with this, cohort studies in highly active and refractory patients, many with prior surgery or neurostimulation, reported clinically meaningful reductions in seizure severity and frequency, with ~ 70% achieving 50–99% seizure reduction and a subset attaining seizure freedom, particularly at doses above 250 mg/day (Aboumatar et al. 2022; Chung et al. 2020; Krauss et al. 2020; Nakamura et al. 2019; Rosenfeld et al. 2021). Padsevonil, designed to target both Synaptic Vesicle Glycoprotein 2 A (SV2A) and GABA_A_Rs, enhances currents preferentially through α1- and α5-containing receptors but showed only moderate efficacy in phase II studies of highly DRE (Niespodziany et al. 2020; Rademacher et al. 2022). Together, these agents illustrate a broader pharmacological shift toward exploiting preserved inhibitory mechanisms beyond classical benzodiazepine-sensitive synapses.

## Modulating Chloride Homeostasis and Tonic Inhibition in DRE

Inhibitory neurotransmission critically depends on the transmembrane chloride gradient, making chloride homeostasis an attractive therapeutic target in epilepsy. Disruption of this gradient, particularly through altered expression of the cation–chloride cotransporters NKCC1 and KCC2, can render GABAergic signalling depolarising and excitatory (Huberfeld et al. 2007; Palma et al., 2006). This “immature” GABA phenotype is prominent in the neonatal brain but re-emerges in epileptogenic tissue, contributing to seizure susceptibility and pharmacoresistance (Ben-Ari 2012; Kaila et al. 2014). The loop diuretic bumetanide inhibits NKCC1 with much higher affinity than KCC2, thereby lowering intracellular chloride and shifting GABA_A_R responses toward hyperpolarisation (Sivakumaran and Maguire 2016). Preclinical studies demonstrate that bumetanide can convert depolarising GABA currents into inhibitory ones and suppress epileptiform activity. Early clinical trials in neonates reported transient reductions in seizure burden when bumetanide was added to phenobarbital; however, efficacy was inconsistent and complicated by ototoxicity, diuresis, hypokalaemia alkalosis, and poor blood–brain barrier penetration (Kaila and Löscher 2022; Soul et al. 2021). Because bumetanide is ionised at physiological pH and actively effluxed from the CNS, high doses are required to achieve central NKCC1 blockade, narrowing its therapeutic window (Töllner et al. 2015). An open-label trial in DRE reported responder rates exceeding 60% after several months of add-on bumetanide therapy, suggesting potential benefit in selected patients (Gharaylou et al. 2019). Nonetheless, systemic toxicity and limited CNS bioavailability remain major obstacles. Current strategies aim to improve bumetanide delivery through prodrugs, lipophilic derivatives, and alternative loop diuretics with enhanced brain penetration (Kaila and Löscher 2022). An alternative approach is to enhance KCC2-mediated chloride extrusion. Preclinical work shows that restoring the phosphorylation state and trafficking of KCC2 can re-establish hyperpolarising GABA signalling, restore benzodiazepine sensitivity, and suppress seizures in models of SE (K. L. Lee et al. 2022a, b). These findings support KCC2 as a disease-modifying target rather than a purely symptomatic one. Beyond chloride, bicarbonate gradients also shape the GABA reversal potential. Because the equilibrium potential for HCO₃⁻ is relatively depolarised, carbonic anhydrase inhibitors such as topiramate, zonisamide, and acetazolamide may contribute to their antiseizure effects by lowering intracellular bicarbonate and shifting E_GABA_ in a hyperpolarising direction (Auer et al. 2020; Shukralla et al. 2022). However, pH modulation also influences other channels, including acid-sensing ion channels, highlighting the interconnected nature of ionic homeostasis in epileptic tissue.

## Pathways to Targeted Therapies

### Spatial Transcriptomic and Structural Characterisation

Recent advances in spatial transcriptomics have given researchers an unprecedented ability to map gene and protein expression with excellent anatomical precision, from single cells down to subcellular compartments. In epilepsy research, these methods are revolutionising our understanding of disrupted neural circuits. By linking molecular changes in one brain region to those in connected regions across epigenetic, transcriptomic, and proteomic levels, spatial approaches allow us to study neurons in their natural network context (J. Lee et al. 2022a, b; Shi et al. 2025). They reveal how hyperactivity in specific neurons can cascade into circuit-wide dysfunction, potentially driven by coordinated genomic and epigenomic programs that underlie epileptogenesis. This kind of context-rich molecular insight opens exciting opportunities to identify new therapeutic targets grounded in neural circuit architecture. Complementing this systems-level perspective, a detailed understanding of how drugs interact with receptors at the molecular level is equally critical. Molecular modelling relies on high-resolution structural data to define receptor architecture and predict how ligand binding reshapes receptor conformation and channel activity (Adelusi et al. 2022). Until recently, structural insight into GABA_A_Rs was lacking, leaving gaps in our understanding of inhibitory pharmacology. Integrating spatially resolved molecular data with precise receptor-level insights provides a powerful framework for connecting circuit dysfunction to pharmacological intervention in epilepsy. Cryogenic electron microscopy (cryo-EM) has now bridged this gap by revealing full-length synaptic GABA_A_R and linking receptor architecture to pharmacological signalling (Kim et al. 2020; Masiulis et al. 2019). Beyond the classical high-affinity benzodiazepine site at the α–γ interface, cryo-EM has uncovered additional low-affinity binding pockets, including a transmembrane site at the α–β interface predicted from functional studies, and distinct sites at the β–α and γ–β interfaces. Functionally, these sites explain the biphasic concentration–response of diazepam. At supratherapeutic levels (> 20 µM), diazepam can potentiate receptors lacking γ2 subunits, effects that are insensitive to flumazenil and mediated by non-classical low-affinity sites rather than the canonical α–γ interface (Ramerstorfer et al. 2011; Wongsamitkul et al. 2017). Although these concentrations are far above therapeutic levels (< 0.3 µM), activation of these sites may contribute to anaesthetic or off-target effects. Cryo-EM also shows that partial agonists nestle deeper within the α–γ pocket than full agonists, providing a structural explanation for differences in efficacy between drugs such as diazepam and bretazenil (Masiulis et al. 2019). Yet, questions remain about whether these low-affinity sites alone can influence behaviour and whether they can be selectively targeted therapeutically.

### Neurosteroid Binding Architecture and Allosteric Control of GABA_A_ Receptors

Structural studies have similarly transformed our understanding of neurosteroid modulation. Early models suggested separate binding sites for neurosteroid activation and allosteric potentiation on the α subunits and at the α–β interfaces (Hosie et al. 2006). Cryo-EM structures of GABA_A_R chimaeras bound to tetrahydrodeoxycorticosterone (THDOC) revealed that both effects arise from a shared transmembrane site (Laverty et al. 2017). In contrast, sulphated neurosteroids, such as pregnanolone sulphate, bind distinct intrasubunit pockets within α subunits, exerting inhibitory effects. More recent work demonstrates that neurosteroids can promote either activation or desensitisation, depending on whether they bind to intersubunit β–α interfaces or intrasubunit β3 domains (Miller et al. 2017; Sugasawa et al. 2020). Whether synaptic and extrasynaptic GABA_A_Rs possess structurally distinct neurosteroid pockets remains unknown, and the lack of subtype-selective neurosteroids limits precision pharmacology. The functional impact of GABA_A_R modulation depends heavily on their regional, cellular, and subcellular distribution. Benzodiazepine sensitivity hinges on a conserved histidine residue in the α1, α2, α3, and α5 subunits, but these subunits are widespread across the brain, so global modulation can produce both therapeutic and side effects (Kim and Hibbs 2021). Circuit-specific understanding is therefore essential. The amygdala illustrates this complexity: its heterogeneous subdivisions and distinct cell types differentially regulate emotional behaviour (Yu et al. 2023). Although most GABA_A_R subtypes are present throughout the amygdala, their localisation varies across nuclei and neuronal classes (Song et al. 2022). In the central amygdala, α5-containing GABA_A_Rs in protein kinase C δ^+^ neurons control anxiety, with knockdown causing anxiogenesis (Botta et al. 2015; Herman et al. 2013). Similarly, α1-containing receptors on corticotropin-releasing factor neurons influence anxiety by modulating excitability (Gafford et al. 2012). However, benzodiazepines non-selectively potentiate all α1–3,5-containing receptors across amygdala circuits, making it difficult to target precise behavioural mechanisms.

### Molecular Regulation of GABA_A_ Receptor Localisation and Synaptic Targeting

Subcellular localisation of GABA_A_Rs is a major determinant of inhibitory strength and is tightly regulated by scaffold proteins, particularly gephyrin, which organizes inhibitory postsynaptic densities (Fig. 3A). Gephyrin forms oligomeric lattice structures beneath the postsynaptic membrane that anchor γ2-containing GABA_A_Rs and stabilize receptor clusters opposite presynaptic release sites (Tretter et al. 2012). Experimental disruption of gephyrin markedly reduces synaptic clustering of α2- and γ2-containing receptors and weakens inhibitory postsynaptic currents, demonstrating that gephyrin-dependent receptor aggregation is essential for efficient inhibitory transmission (Lévi et al. 2004). Importantly, alterations in gephyrin organization have been reported in TLE. In pilocarpine-induced status epilepticus models in rodents, gephyrin clusters become fragmented and reduced in density within hippocampal CA1 and dentate gyrus neurons, leading to impaired synaptic anchoring of GABA_A_Rs and decreased inhibitory synaptic strength (González et al. 2013; Thind et al. 2010). Similar reductions in gephyrin expression and postsynaptic clustering have been observed in hippocampal tissue resected from patients with drug-resistant TLE, suggesting that scaffold destabilisation contributes to impaired inhibitory signalling in human disease (Fang et al. 2011). Receptor localisation also depends on subunit composition and intracellular targeting motifs that regulate interactions with scaffold proteins and determine membrane mobility. In hippocampal pyramidal neurons, α5-containing GABA_A_Rs are predominantly extrasynaptic and mediate tonic inhibition, although they can also participate in phasic signalling under certain conditions (Magnin et al. 2019). Circuit-specific targeting further shapes receptor distribution: in mouse hippocampal networks, vasoactive intestinal polypeptide and calretinin interneurons synaptically recruit α5-containing receptors onto somatostatin interneurons, forming disinhibitory microcircuits that regulate anxiety-related behaviours (Magnin et al. 2019). In epileptic networks, alterations in receptor subtype localisation, including increased expression of α4-containing receptors and reduced synaptic anchoring of α1-containing receptors, have been observed in pilocarpine and kainate rodent epilepsy models as well as in resected hippocampal tissue from DRE patients, suggesting that pathological redistribution of receptor subtypes contributes to altered inhibitory dynamics (Brooks-Kayal et al. 1998; Peng et al. 2004). The functional diversity of GABA_A_Rs is further expanded through alternative splicing and post-translational modification, which regulate receptor trafficking and pharmacological responsiveness. The γ2 subunit exists in long (γ2L) and short (γ2S) splice variants that differ by eight amino acids within the intracellular loop, influencing receptor trafficking, channel kinetics, and zinc sensitivity (Boileau et al. 2010). The γ2L isoform contains a phosphorylation site at S343 that modulates receptor insertion and synaptic retention through kinase-dependent signalling pathways (Lorenz-Guertin et al. 2018). These findings indicate that dynamic regulation of receptor scaffolding and post-translational signalling pathways may play a critical role in the progression of inhibitory dysfunction in DRE.

### Auxiliary Proteins and Trafficking Control

Native GABA_A_Rs function within large macromolecular complexes that include scaffolding proteins, trafficking regulators, and auxiliary subunits that collectively control receptor localisation, stability, and pharmacological sensitivity (Schmerl et al. 2022). Central to this network is gephyrin, which anchors γ2-containing receptors at inhibitory synapses and regulates receptor surface diffusion through dynamic scaffold assembly (Tretter et al. 2012). Gephyrin recruitment to synapses requires the adaptor protein collybistin, which links gephyrin to the postsynaptic membrane. Mutations in the ARHGEF9 gene encoding collybistin disrupt gephyrin clustering and produce epilepsy and intellectual disability in humans, highlighting the essential role of the gephyrin scaffold in maintaining inhibitory circuit stability (Papadopoulos et al. 2007; Wang et al. 2018). Disruption of this gephyrin–collybistin complex has also been observed in mouse models of epilepsy, where impaired receptor anchoring leads to reduced inhibitory synaptic strength and increased neuronal excitability. Additional auxiliary proteins further refine receptor trafficking and synaptic targeting. GARLH4 (GABA_A_R regulatory Lhfpl4) stabilises γ2-containing receptors at synapses by linking them to neuroligin-2, a postsynaptic adhesion molecule critical for inhibitory synapse formation. Genetic deletion of GARLH4 in mouse knockout models significantly reduces synaptic receptor clustering and inhibitory synapse density, demonstrating its importance for maintaining inhibitory synaptic architecture (Tipton and Russek 2022; Wu et al. 2018). In contrast, δ-containing receptors which mediate tonic inhibition exclude GARLH4 and γ2, remaining largely extrasynaptic (Lu et al. 2020). Dysregulation of tonic inhibition mediated by δ-containing receptors has been reported in kainate and pilocarpine epilepsy models, suggesting that altered auxiliary protein interactions may contribute to network hyperexcitability. Intracellular trafficking of GABA_A_Rs is also controlled by proteins that regulate receptor maturation and forward transport. Clptm1 negatively regulates receptor surface expression by retaining GABA_A_Rs within the endoplasmic reticulum, thereby modulating both phasic and tonic inhibitory signalling (Chen et al. 2025). Dysregulation of receptor trafficking pathways has been implicated in seizure-induced receptor internalisation and reduced synaptic inhibition observed during prolonged seizures. More recently, Shisa7 was identified as the first auxiliary subunit specific to GABA_A_Rs. Shisa7 co-localises with gephyrin at inhibitory synapses and regulates receptor trafficking, channel kinetics, and benzodiazepine pharmacology. In Shisa7 knockout mice, inhibitory postsynaptic current decay is prolonged and the behavioural efficacy of diazepam is significantly reduced, demonstrating that auxiliary proteins can directly influence benzodiazepine sensitivity and inhibitory synaptic function (Castellano et al. 2022). These discoveries are prompting a shift in drug development toward “thinking outside the pore.” For example, artemisinin derivatives have been shown to bind gephyrin and destabilise α1- and α2-containing receptor clusters in neuronal cultures and mouse models, demonstrating that scaffolding proteins themselves can be pharmacologically targeted (Kasaragod et al. 2019). By integrating receptor architecture with circuit-specific localisation and auxiliary protein regulation, future therapies may achieve greater precision by selectively modulating defined GABA_A_R populations and their associated regulatory complexes, an approach that may ultimately improve treatment outcomes in DRE. Table 2 summarises the potential therapeutic targets discussed and their mechanisms of action.

**Table 2:** Mechanistic strategies targeting GABAergic dysfunction in drug-resistant epilepsy

Mechanistic target	Preclinical findings	Clinical findings	Limitations	References
Non-classical positive allosteric modulation of GABA_A_ receptors (Cannabidiol; CBD)	CBD acts as a positive allosteric modulator of α-containing GABA_A_ receptors, with greater efficacy at α2β2/3γ2L assemblies than β1-containing receptors. It potentiates GABA-evoked currents without binding the classical benzodiazepine site, indicating modulation through distinct receptor interfaces that may restore inhibition in circuits with benzodiazepine-insensitive receptor remodelling.	Observational and randomised trials show clinically meaningful seizure reductions in severe epileptic encephalopathies. In Dravet syndrome, adjunctive CBD (~ 20 mg/kg/day) reduced seizures by ~ 50% vs. ~ 27% with placebo. In Lennox–Gastaut syndrome, seizure frequency decreased by ~ 44% vs. ~ 22% with placebo.	Seizure freedom remains uncommon. CBD has low oral bioavailability, extensive first-pass metabolism, and pharmacokinetic interactions with clobazam, complicating the interpretation of efficacy. Its multimodal pharmacology also obscures the specific contribution of GABAergic modulation.	(Bakas et al. 2017; Berg et al. 2024; Bialer and Perucca 2020; Bonanni et al. 2023; Devinsky et al. 2019; French et al. 2017; Gunning et al. 2021; Koo et al. 2020; Millar et al. 2020; Na and Lee 2025; Ruffolo et al. 2018)
Extrasynaptic GABA_A_ receptor potentiation and tonic inhibition (Neurosteroids)	Neurosteroids positively modulate GABA_A_ receptors via transmembrane binding sites on α- and β-subunits, preferentially potentiating δ- and α6-containing extrasynaptic receptors that mediate tonic inhibition. In rodent seizure models, allopregnanolone enhances inhibitory currents and suppresses epileptiform activity, even when synaptic receptors are downregulated. Ganaxolone, a 3β-methylated analogue, retains neurosteroid activity while preventing metabolic back-conversion.	Early studies suggested brexanolone may facilitate withdrawal of anaesthetic anticonvulsants in super-refractory status epilepticus, but a phase III trial failed to show superiority over placebo. Ganaxolone trials in refractory epilepsy report modest seizure reductions (~ 21% vs. ~ 10% placebo) with good tolerability.	Clinical efficacy remains modest and variable, and neurosteroids lack strong receptor subtype selectivity. Significant translational gaps persist between robust preclinical efficacy and clinical outcomes.	(Kanes et al. 2016; Lappalainen et al. 2017; Laxer et al. 2000; MacKenzie and Maguire 2013; Miziak et al. 2020; Rogawski et al. 2013; Rosenthal et al. 2017; Treviño et al. 2025; Walsh et al. 2025)
Multimodal GABAergic modulation by newer antiseizure medications (Stiripentol, Cenobamate, Padsevonil)	Stiripentol prolongs GABA_A_ receptor channel opening and preferentially modulates α3- and δ-containing receptors. Cenobamate combines voltage-gated sodium channel inhibition with non-benzodiazepine positive allosteric modulation of α1–6 GABA_A_ receptors. Padsevonil was designed as a dual-target agent combining SV2A binding and GABA_A_ receptor modulation.	Cenobamate shows strong efficacy in focal epilepsy, with > 50% seizure reduction and 11–26% seizure-freedom rates in trials; real-world cohorts report ~ 70% responder rates in refractory populations. Stiripentol is approved for Dravet syndrome. Padsevonil showed only moderate efficacy in phase II DRE trials.	Mechanistic specificity remains incomplete. Sedation, drug interactions, and variable patient response limit effectiveness, and padsevonil development stalled due to insufficient benefit in highly drug-resistant populations.	(Aboumatar et al. 2022; Bialer and Perucca 2026; Chung et al. 2020; Fisher 2009; Krauss et al. 2020; Nakamura et al. 2019; Niespodziany et al. 2020; Quilichini et al. 2006; Rademacher et al. 2022; Rosenfeld et al. 2021; Strzelczyk et al. 2025; Zhang et al. 2021)
Restoration of chloride homeostasis and GABA polarity (Bumetanide)	Experimental models show NKCC1 upregulation and KCC2 downregulation, leading to elevated intracellular chloride and depolarised GABA responses. Bumetanide inhibits NKCC1, reducing intracellular chloride and restoring hyperpolarising GABAergic inhibition, suppressing epileptiform activity in rodent and slice models.	Clinical studies in neonatal seizures report transient seizure reduction when bumetanide is combined with phenobarbital. An open-label DRE study reported > 60% responder rates after several months of adjunctive therapy.	Translation is limited by poor BBB penetration, ototoxicity, electrolyte disturbances, and a narrow therapeutic window, restricting long-term use.	(Ben-Ari 2012; Bolla et al. 2025; Gharaylou et al. 2019; Huberfeld et al. 2007; Kaila and Löscher 2022; Kaila et al. 2014; Palma et al., 2006; Römermann et al. 2017; Sivakumaran and Maguire 2016; Soul et al. 2021; Töllner et al. 2015)
Enhancement of KCC2-mediated chloride extrusion	Restoring KCC2 phosphorylation and membrane trafficking enhances chloride extrusion and re-establishes hyperpolarising GABA responses. In rodent models of status epilepticus, KCC2 normalisation restores benzodiazepine sensitivity, implicating chloride dysregulation in the development of pharmacoresistance.	Direct KCC2-targeted therapies remain experimental and have not yet advanced to clinical trials.	The lack of clinically viable KCC2 activators and the limited understanding of long-term network effects currently limit translation.	(K. L. Lee et al. 2022a, b)
pH- and bicarbonate-dependent modulation of GABA signalling (Carbonic anhydrase inhibitors)	Carbonic anhydrase inhibition lowers intracellular bicarbonate, shifting E_GABA_ toward hyperpolarisation and strengthening inhibitory signalling. pH changes also regulate neuronal excitability via acid-sensing ion channels and related conductance.	These agents are widely used clinically as antiseizure medications across multiple epilepsy syndromes.	Mechanistic effects are non-specific, and seizure control remains incomplete in many DRE patients.	(Auer et al. 2020; Biton 2007; Costa et al. 2010; Shukralla et al. 2022)
Receptor trafficking and inhibitory synapse scaffolding (gephyrin, collybistin, GARLH4, Shisa7)	Epilepsy models show gephyrin scaffold fragmentation and reduced synaptic clustering of GABA_A_ receptors, weakening inhibitory transmission. Disruption of collybistin or GARLH4 impairs receptor anchoring and inhibitory synapse formation.	ARHGEF9 (collybistin) mutations cause epilepsy and intellectual disability in humans, demonstrating the clinical relevance of inhibitory synapse scaffolding defects.	Pharmacological targeting of scaffold proteins could disrupt widespread inhibitory synapses, creating safety and specificity concerns.	(Burdina et al. 2025; Castellano et al. 2022; Chen et al. 2025; Fang et al. 2011; González et al. 2013; Kasaragod et al. 2019; Lévi et al. 2004; Lu et al. 2020; Papadopoulos et al. 2007; Schmerl et al. 2022; Soykan et al. 2014; Thind et al. 2010; Tipton and Russek 2022; Tretter et al. 2012; Wang et al. 2018; Wu et al. 2018)

## Emerging Experimental Models

Recent advances in stem-cell technology have enabled the development of human-derived experimental models that complement traditional rodent epilepsy models and provide new platforms for studying DRE. One major approach involves patient-derived induced pluripotent stem cells (iPSCs), which can be reprogrammed from somatic cells and differentiated into neuronal populations carrying the patient’s genetic background. For example, neurons generated from individuals with Dravet syndrome (caused by SCN1A mutations) display spontaneous bursting activity and increased sodium currents, demonstrating intrinsic hyperexcitability and validating the use of iPSC-derived neurons to model epileptic phenotypes in vitro (Liu et al. 2013). Similarly, iPSC-derived neurons from Dravet patients show impaired action potential generation in GABAergic neurons, supporting the hypothesis that interneuron dysfunction contributes to epileptogenesis (Higurashi et al. 2013). More recent work has expanded these systems to patient-specific iPSC lines carrying epilepsy-associated mutations, enabling transcriptomic analyses that reveal disease-relevant gene expression changes and providing a platform for personalised therapeutic screening (Wu et al. 2025). Three-dimensional brain organoids and assembloids represent a further development of stem-cell-based models, allowing partial reconstruction of human cortical architecture and network activity. For instance, cortical organoids derived from patients carrying SCN2A channelopathy variants exhibit increased neuronal spiking and network bursts, reproducing seizure-like hyperexcitability and enabling evaluation of differential responses to anti-seizure drugs using multielectrode arrays (Yang et al. 2025). Organoid models have also been used to investigate developmental and epileptic encephalopathies, where CRISPR-engineered or patient-derived organoids reveal disrupted neural differentiation and altered signalling pathways underlying severe epilepsy phenotypes (Steinberg et al. 2021). Together, iPSC-derived neuronal cultures and organoid systems provide powerful human-specific platforms for modelling epileptogenesis, network hyperexcitability, and pharmacological responses, offering promising tools for mechanistic studies and precision medicine approaches in DRE research.

## Conclusion

DRE persists because seizures emerge not from a single molecular failure, but from coordinated disruption of GABA_A_R composition, trafficking, chloride homeostasis, and circuit integration that progressively destabilise inhibitory control. Altered subunit expression, impaired receptor localisation, and pathological shifts in NKCC1/KCC2 balance collectively transform GABAergic signalling from a restraining force into a contributor to network hyperexcitability. These multilayered changes help explain why many conventional anti-seizure drugs lose efficacy in chronic epilepsy and why restoring inhibition remains a central therapeutic challenge. Emerging tools such as spatial transcriptomics, proteomics, and cryo-EM now enable mapping of how synaptic and extrasynaptic GABA_A_R subtypes and their auxiliary proteins are reorganised across epileptic networks. The next challenge is to translate these molecular maps into therapy by developing subtype-selective ligands, neurosteroid analogues, and compounds targeting receptor-associated proteins such as GARLH4, Clptm1, and Shisa7, while simultaneously restoring chloride gradients by modulating KCC2/NKCC1. Linking patient-specific GABAergic signatures to precision pharmacology may ultimately shift DRE treatment from non-specific suppression towards mechanism-guided control of pathological inhibition.

## Data Availability

No datasets were generated or analysed during the current study.
